# Regional assemblage and the spatial reorganisation of health and care: the case of devolution in Greater Manchester, England

**DOI:** 10.1111/1467-9566.12867

**Published:** 2019-02-13

**Authors:** Colin Lorne, Ruth McDonald, Kieran Walshe, Anna Coleman

**Affiliations:** ^1^ Department of Health Services Research and Policy London School of Hygiene & Tropical Medicine London UK; ^2^ Alliance Manchester Business School University of Manchester Manchester UK; ^3^ Centre for Primary Care University of Manchester Manchester UK

**Keywords:** geography, government/state, National Health Service (NHS), healthcare systems, space/spatial factors, politics

## Abstract

In this paper, we examine how space is integral to the practices and politics of restructuring health and care systems and services and specifically how ideas of assemblage can help understand the remaking of a region. We illustrate our arguments by focusing on health and social care devolution in Greater Manchester, England. Emphasising the open‐ended political construction of the region, we consider the work of assembling different actors, organisations, policies and resources into a new territorial formation that provisionally holds together without becoming a fixed totality. We highlight how the governing of health and care is shaped through the interplay of local, regional and national actors and organisations coexisting, jostling and forging uneasy alliances. Our goal is to show that national agendas continued to be firmly embedded within the regional project, not least the politics of austerity. Yet through keeping the region together as if it was an integrated whole and by drawing upon new global policy networks, regional actors strategically reworked national agendas in attempts to leverage and compete for new resources and powers. We set out a research agenda that foregrounds how the political reorganisation of health and care is negotiated and contested across multiple spatial dimensions simultaneously.

## Introduction

The political reorganisation of health and care is both temporal *and* spatial. Health policy scholars commonly incorporate a longitudinal approach examining the former (for instance, Powell 2003). Historically sensitive work considers how ‘sedimented layers’ of past healthcare reforms continue to unevenly shape contemporary governing practices (Jones 2017). However, the spatial dimensions of restructuring are often ignored or downplayed, despite appeals otherwise (Moon 1990, Moon and Brown 2000). Where scholarship has engaged with the political construction of space shaping healthcare reform, there is a tendency for analysis to privilege a single spatial dimension as *the* definitive concept for examining historical or contemporary changes (Jessop *et al*. 2008). Understandably, this scholarship often responds to policy agendas which either explicitly or implicitly adopt geographic concepts such as place (Hammond *et al*. 2017, Prince *et al*. 2006), localism (Allen 2006) or community (Moon 1990). Perhaps unsurprisingly, studies tend to find the latest spatialised policy rhetoric often does not meet with reality.

In this paper, we build on spatially sensitive accounts of health and care reform to examine the remaking of a region through devolution. We consider how reforms are negotiated and contested across multiple spatial dimensions simultaneously. To do so, we take inspiration from critical scholars adopting ideas of assemblage to understand the remaking of regions (Allen and Cochrane 2007, 2010) and public services (Newman and Clarke 2009). As we discuss below, we understand an assemblage as the gathering together of heterogeneous and often ill‐fitting elements into a provisional socio‐spatial formation. Assemblages never become a totality, but require work to sustain them and keep them together or they fragment and fall apart. Thus, an assemblage, in our case a region, is made through ongoing practices of assembling new actors, resources or policies and disassembling others to arrange health and social care systems and services into a new meaningful, if not necessarily coherent or conflict‐free, project.

To illustrate how ideas of assemblage help us understand the practices and politics of reorganising health and care, we use the high‐profile example of health and social care devolution in the metropolitan region of Greater Manchester, England.^1^ The Greater Manchester project incorporates elements of reform which happen across different spatial dimensions which are subject to international scholarly attention. Devolution involves the rescaling of power through transferring function and responsibilities from national to subnational levels. Changes incorporate place‐based integration of health and care, in spite of the political and technical questions raised through bringing universal health care and means‐tested social care together. Integrated care models draw upon learning from trans‐national health policy networks as the latest ideas are mobilised by a seemingly ever‐expanding circuit of policy experts. This all takes place within a complex, fragmented institutional landscape of public, private and voluntary sector organisations interested in the organisation, provision, regulation and improvement of health and social care as well as infrastructure such as buildings, medicines and technologies. Devolution affects 2.8 million people in the region, many of whom are unpaid or underpaid carers or work for the National Health Service (NHS) as one of the biggest employers in Greater Manchester. Yet reform is taking place in an age of centrally driven ‘super‐austerity’ (Gray and Barford 2018, Lowndes and Gardner 2016). Local authority budgets have been cut and NHS healthcare budgets witnessed the lowest increase in its 70‐year history. Given the uncertainties surrounding the project, academics have termed it the ‘Greater Manchester experiment’ (Walshe *et al*. 2016), although think‐tanks such as the King's Fund have begun to position Greater Manchester as an exemplar integrated care system. Fundamentally, the remaking of health and care in the region is a political process.

The paper proceeds as follows. We outline our conceptual focus on space and assemblage thinking in relation to sociological scholarship concerned with health and care. We then situate our case study and our research methodology. Framing Greater Manchester as a regional assemblage, we examine how the restructuring of health and care holds together through the interplay of local, regional and national actors, organisations and agencies coexisting, jostling and forging uneasy alliances. We highlight the ongoing work required to sustain fragile relations within the region. Emphasising the heterogeneity of the assemblage, we outline how attempts by national actors to embed their agendas in the region were more‐or‐less successful and connectedly, how these were reworked by regional actors in attempts to leverage new resources and powers. A discussion of our contributions concludes the paper, setting out a research agenda foregrounding how the political reorganisation of health and care is negotiated and contested across multiple spatial dimensions simultaneously.

## Conceptual approach: space and assemblage

Despite the prevalence of geographic ideas within health policy, spatially inflected analysis has often experienced a rather marginalised position within sociological studies of healthcare reform (Moon 1990). Not unconnectedly, it has been some time since Robin Kearns (1993) appealed to geographers to engage with a renewed geography of health that moved beyond medical geography and engaged more fully with social theory, a call that continues to resonate. Brown *et al*. (2017) capture the plurality of subsequent scholarship situated between sociology of health and social geography. Of course, various sociological studies have utilised geographic concepts to examine how space is entangled within care practices, for instance, embodied care work and spaces of home (England and Dyck 2011), how place and care are co‐produced (Ivanova *et al*. 2016) and the architectural spaces of healthcare buildings (Martin *et al*. 2015). Nonetheless, while there have been a number of spatially sensitive sociological studies of health care across a range of scales, geographic contributions within healthcare policy and reform remain under‐valued.

There is a historical, if now somewhat outmoded, tendency among sociologists to treat space as Cartesian and objective, waiting to be filled with meaning to become a place (Urry 2001). We insist, however, that space is not ‘abstract geometries’ (cf. Gieryn 2000: 465), but instead integral to the ways in which places, be that cities, regions or continents, are constructed. Put most simply, space is socially produced. We can understand regions as constituted through the juxtaposition of multiple, interweaving social relations, some of which may be fleeting, others more enduring, some relationships may be more local, others seemingly global (Massey 2005). This incorporates an awful lot, far more than a concern for health and social care systems and services. Yet it provides a helpful entry point into thinking about how the remaking of regional health and care is not merely what happens within the delineated territorial boundaries of a healthcare system. Space is politically contested and struggled over. And as social‐spatial entities, Kivelä and Moisio (2017: 30) remind us, health and care systems are ‘neither static nor politically neutral’ but instead a key dimension of the ongoing spatial transformation of the state.

Ideas of assemblage are one approach that helps examine the multiple socio‐spatial dimensions of such transformation. Frequently associated with the writings of Deleuze and Guattari (1988), scholarship concerned with assemblages has witnessed growing interest among social scientists in recent years. Assemblage has been used in a variety of ways, from a non‐conceptual descriptive device through to an ontological orientation for thinking about the emerging composition of relations in the world (Anderson *et al*. 2012). Given the rather dense theoretical language associated with these ideas, we broadly define an assemblage as a provisional gathering together of diverse and often ill‐fitting elements into a socio‐spatial formation, without becoming a fixed totality. Our interpretation enables us to examine the ‘double dynamics of solidity and fragility’ as heterogeneous elements of people, organisations, policies and resources are brought together into a meaningful order for some time (Newman and Clarke 2009: 15). To sustain assemblages, work is required to smooth over fractures and contradictions otherwise the assembled formation is at risk of breaking apart and failing. With health and care increasingly shaped by a multitude of public, private and voluntary sector organisations, assemblage thinking provides a useful framing for examining the practices of orientating and stabilising health and care systems and services between conflicting or diverging elements. It pays attention to the practices of unmaking and remaking as an assemblage mutates, new policies or political actors are introduced or relationships cannot be sustained and things fall apart.

Assemblages thus require labour to hold them together as new elements become attached or alliances are not maintained (McFarlane 2011). This is the *assembling* and *disassembling* or *dismantling* work involved in the ongoing formation of assemblages. This approach focuses on the ongoing relationships between the whole of a socio‐spatial formation and its parts. More specifically, it encourages consideration to be paid to the associations and alliances of the different elements forming assemblages rather than their individual properties, addressing how these relationships adapt in particular configurations and change over time. This entails a view of agency which recognises the intersubjectivity of meaning and action. Agency is understood as distributed, made possible through the associations of relations that make up the assemblage, rather than rooted in the capabilities of specific individuals. With an emphasis on process, assemblage thinking blurs the structure/agency dualism, paying attention to how even seemingly well‐ordered formations are open to the unexpected and contingent. That agency is an emergent property of an assemblage does not convey that power is evenly or randomly dispersed, but instead that some of the points at which the trajectories of groups and individuals intersect are busier and more significant than others (Bennett 2005). Thus, no central power or single individual can determine the functions of an assemblage. Rather, we focus on the ongoing relations of assembling, stabilising and disassembling that constitute a particular matter of concern.

Ideas of assemblage have been used to understand the political remaking of regions. Allen and Cochrane (2007, 2010) adopt this approach to conceptualise regions as made of a multitude of variously ‘local’, ‘regional’, ‘national’ and ‘global’ actors, organisations and agencies that are co‐present and embedded within regions, coexisting, jostling and forming uneasy alliances in different ways:

Some of this interplay takes place indirectly by authorities *reaching into* the politics of regions and localities in an attempt to steer and constrain agendas; some of it operates in a more direct fashion by *drawing within close reach* those that are able to broker and influence decisions; whilst other forms of mediated interaction *reach out* beyond the region or locality to shape events within. (Allen and Cochrane 2010: 1075; original emphasis)

The institutional hierarchical organisation of the state, which invariably gets labelled ‘local’ or ‘national’ is not abandoned, but there is a recognition that spaces such as regions are constructed through the interplay of overlapping, entangled and unstable negotiations of power that hold together rather than exist at a particular spatial scale *a priori*. Our reading does not eschew the idea that decision‐making may be rescaled from national to regional or local institutions, or that we might talk about operating within a highly centralised state *per se*. Rather, it encourages attention to the ongoing arrangement of different local, regional or national actors and how they interact and intersect. Understanding regional assemblages in this way helps us examine how certain national actors are able to make their presence felt to govern health and care in a region, while others may be less successful and displaced, or to consider how 'global' management consultants are drawn upon by regional bodies in an attempt to rework national priorities.

Our reading of assemblage provides a mode of enquiry for examining the political remaking of health and care in a devolved region. It helps us appreciate how elements of health and social systems retain a relative degree of autonomy without the region being reducible to discrete parts. It allows us to consider how the integrating of different elements fit together more‐or‐less successfully over time. It illustrates that the governing of a political formation is a provisional accomplishment contingent on the co‐functioning of new policies, organisations and budgets mixing with the residual fragments of previous re‐disorganisations cutting across spatial scales. This foregrounds how seemingly minor events or technical procedures are connected to the spatial politics of health and care restructuring which can sometimes lead to surprising and unexpected outcomes. We elaborate on this in our presentation of empirical findings and discussion, after situating our case study and methodology in the following sections.

## Devolving health and social care in Greater Manchester, England

Located in North West England, Greater Manchester is a metropolitan region with a population of approximately 2.8 million. It is comprised of 10 local authority areas with a history of working together following the abolition of the metropolitan county councils by the Thatcher government in the 1980s. Leaders in Greater Manchester have long positioned the region as a ‘proto‐devolution experiment’ strategically using its history of voluntary collaboration between predominantly Labour‐led local authorities to emphasise their ability to work with successive central governments (Haughton *et al*. 2016: 10). By 2011, the Greater Manchester Combined Authority was formed out of the 10 local authorities and by 2014 a devolution deal was agreed with central government. This gave the region new, if highly constrained, controls over issues such as transport, housing and planning, conditional on an elected Metro Mayor and privileging agglomerative urban economic growth (Haughton *et al*. 2016). The largely secretive agreement set a precedent for a patchwork of subsequent deal‐based devolution settlements across England, heavily steered by central government with an absence of a solid constitutional basis or public debate (Ayres *et al*. 2018).

The 2014 deal provided the catalyst for a second separate health and social care devolution deal for Greater Manchester, the principal focus of this paper (hereafter, ‘devolution’ refers to health and social care devolution unless otherwise stated). Devolution was an initiative to remake Greater Manchester *in its entirety* out of the fragments and failures of previous health and care reforms. This included the contentious *Health and Social Care Act 2012* intended to promote market‐competition and the imperative for NHS trusts to compete with one another as well as private providers and for commissioners to spend their resources by choosing between providers (Jones 2017). Through devolution, the region became the target for a set of policy and political outcomes established behind‐the‐scenes by an elite group of political and managerial actors from central government, local government and the English NHS. The content of this agreement was set out in a *Memorandum of Understanding* (MoU) in February 2015 (Association of Greater Manchester Authorities [AGMA]/NHS England/Greater Manchester Association of Clinical Commissioning Groups [GMACCGs] 2015). By December 2015, a strategic plan for the region known as *Taking Charge of our Health and Social Care in Greater Manchester* was produced (Greater Manchester Combined Authority and NHS in Greater Manchester 2015) and plans for each of the 10 localities followed which broadly aligned with *Taking Charge*.

The aims of devolution were that the health and wellbeing of Greater Manchester's population had to improve amidst high levels of social and health inequalities, health and care services were to become integrated despite health care being principally free at the point of delivery while social care means‐tested, clinical sustainability of services was required, and underpinning all this, the region had to achieve ‘financial sustainability’. Greater Manchester was to work together to close a ‘financial gap’ of £2bn by 2020/1 despite the slowest increase in NHS funding in its history as well as being heavily and unevenly impacted by local government austerity (Gray and Barford 2018). In return, the Greater Manchester deal offered certain ‘internally delegated’ NHS commissioning functions alongside the budgetary responsibility for delivering health and care within the allocated resources. A £450m ‘Transformation Fund’ was agreed to support the changes. On first sight, the extent of ‘devolution’ on offer was highly constrained.

Devolution went live in April 2016. It has been governed by the Greater Manchester Health and Social Care Partnership (Partnership, hereafter), formed out of the then‐37 statutory organisations.^2^ This was originally made up of 10 local authorities, 12 clinical commissioning groups (clinically led organisations responsible for purchasing healthcare services) and 15 NHS hospitals, mental health and community service provider organisations (Foundation Trusts and Trusts) that provide services at least partially within the region. The Partnership also includes representatives from primary care, the voluntary sector and police and fire services. A substantial non‐statutory Partnership Team was established to coordinate changes across Greater Manchester, including the Chief Officer of the Partnership. Significantly, the Chief Officer was employed by NHS England (an influential national arm's length body which has the responsibility to commission some healthcare services and largely steers national health policy on behalf of the Department for Health). Devolution introduced a new layer of decision‐making and control within an already complex organisational landscape, albeit with the regional Partnership Team having only limited formal powers (Checkland *et al*. 2015).

## Methods

Methodologically, assemblage thinking encourages an openness to the unexpected, paying attention to the multiple, uncertain and non‐linear dynamics of making and enacting policy (Baker and McGuirk 2017). Through broadly social‐constructivist and relational understandings of the world, assemblage thinking examines the circulating, translating and reassembling of policy as it becomes embedded in particular places. Advocates encourage researchers to embrace an ‘experimental’ approach which exposes researchers to the uncertainty inherent in processes as complex as reorganising health and care (Anderson *et al*. 2012). There is no methodological blueprint to follow when working with the ideas of assemblage (Allen 2011) so there is a need to balance the tension between careful project research design and attending to unforeseen events, divergent trajectories and new connections (McCann and Ward 2012). This, we suggest, was particularly helpful for researching devolution in real time, not least given its rather speculative, loosely defined origins.

We adopted a qualitative ethnographic approach for our research which took place between the autumns of 2015 and 2017. The focus of the study was to understand the development of the devolution project, the changing governance and organisational arrangements and early changes to services. The themes of subsidiarity, efficiency and integration were identified from the *MoU* guiding the initial direction of research. Data were gathered from observing more than 343 hours of meetings (Greater Manchester‐wide and in localities). Fifty semi‐structured interviews were conducted with senior NHS managers and clinicians, local authority and combined authority staffs, representatives from the voluntary sector as well as management consultancies brought into the region. We collated documentary sources and attended a range of public and private events on devolution.

Observational notes were guided by events arising within meetings, structured around the agenda of the day. Circulated slides and meeting papers were read beforehand. The atmosphere was described, debates and discussions noted and major tensions drawn out in concluding notes. We found ourselves travelling between meetings with managers on occasions and holding conversations after meetings finished. While these insights are not cited directly, they informed our understanding of the ‘behind‐the‐scenes’ work involved in remaking health and care. This helped shape interview questions. Given the breadth of issues arising, informants would often be prompted by recent events that the two lead qualitative researchers had themselves witnessed in meetings. Interviews tended to last approximately 1 hour, although some were closer to half an hour and others more than two. They were mostly held in person in the offices of managers across Greater Manchester. Full observation notes were typed up, enabling sharing of data with other members of the study team. Interviews were transcribed verbatim. Combined data sources were inductively and deductively coded using NVivo v11 and organised thematically. The team (comprising all authors) iteratively reviewed the coding framework and emerging themes at regular team meetings and stakeholder meetings were held with senior managers leading the devolution project to discuss emerging findings throughout.

Therefore, we use the case study of health and social care devolution in Greater Manchester to illustrate how the conceptual approach of assemblage thinking helps us understand the spatial reorganisation of health and care. We examine the work involved in keeping the new regional formation together amidst the coexistence of diverse logics and diverging agendas for the organisation of health and care in Greater Manchester. We focus on how the regional assemblage is shaped by seemingly distant actors and organisations more‐or‐less successfully making their presence felt in the region, distorting the coherence of the new ‘devolved’ territorial entity. We outline how efforts in Greater Manchester to present the assemblage as a unified whole and make new connections elsewhere were strategically used to embed regional agendas nationally in attempts to leverage and compete for new resources and powers.

## Greater Manchester taking charge together – and staying together?

Assemblages never fully cohere into a totality; rather they hold together in more‐or‐less solid and fragile ways *as if* they are whole. The remaking of Greater Manchester through devolution was shaped by this dynamic of solidity and fragility. The project relied on managing consensus among a highly fragmented set of relationships between different organisations each with their own budgets, agendas and oversight mechanisms. Fragility existed right from the early stages when news of the full content of the devolution deal leaked to the local press in advance of the official announcement. A senior manager involved recounts:

The production of the MoU [Memorandum of Understanding] itself back in February last year was done in a ridiculously short period of time, I think we agreed to do it on something like 5th February and we had it done about 3 weeks later, and it was then leaked to the press, and we had a lot of work to do in a 24 hour period to make sure that a lot of CCGs and others who were pretty spooked by the whole thing didn't then jump ship. So a lot of work had to be done to some extent reactively because of the leaking to make sure that the whole thing didn't fracture and fragment’. (Core team member, ID18, August 2016; interview)

Keeping the regional assemblage together was contingent on all local organisations agreeing to stay on‐board with changes that were, at the time, loosely defined. Potential tensions associated with restructuring local commissioning, for instance, threatened to fragment the project from the offset. Concerns were mitigated behind closed doors and fragmenting relationships repaired sufficiently to ensure stability across Greater Manchester. This was the first of many issues that risked destabilising the region as devolution policy began to take hold.

Assembling health and care devolution contained a multitude of contradictions, conflicts and tensions. Some actors were more willing to pursue the changes than others. While health and care in the region was to be remade *as a whole*, there was no one singular Greater Manchester. Uneven legacies, variations and political differences continued to register. Where some parts of the region had been successful at winning national funding for recent NHS pilot projects, other parts were at risk of their local hospital failing. Left wing councillors coexisted awkwardly alongside powerful Chief Executives. Bringing together local elected politicians and local health commissioning was one such example of potentially ill‐fitting elements drawn together into new arrangements:

I'm worried about the future politicisation of health; it worries me. Local authorities see everything as political, with elected members on [local integrated commissioning] boards, what are the implications? What about the role of the elected Greater Manchester Mayor who has no statutory responsibility for health? (NHS provider manager, ID71, May 2017; meeting observations)

For NHS managers, devolution and the integration of health and care were destabilising as new political actors were drawn into decision‐making who had previously not been. This included the new Greater Manchester Mayor who formally, at least, had no influence on the NHS. We dispute the notion that NHS decision‐making was hitherto apolitical. Yet, despite the existence of health scrutiny policies for many years, the new assemblage of actors, many of whom had long been present in the region, were brought together into very different sets of relationships. This generated new uncertainty.

Previously agreed decisions that once helped secure the devolution deal became less secure. *Healthier Together* was an initiative intended to ‘rationalise’ certain A&E, acute medicine and general surgery services across fewer specialist sites in Greater Manchester to ensure equality of provision. While all local CCGs in the region voted unanimously for the changes prior to devolution, it was politically contentious and subsequently legally challenged by local clinicians seeking to ‘*Keep Wythenshawe Special*’ (Segar *et al*. 2016). Their challenge was unsuccessful. Yet in light of devolution which now incorporated a whole host of similar acute service changes having new financial implications, questions over altering the yet‐to‐be‐implemented *Healthier Together* decision resurfaced. Unpicking *Healthier Together* was firmly closed down by key figures in Greater Manchester, despite sitting awkwardly, as they insisted that it cannot be seen to be challenged and instead must fit into the new regional arrangements.

I'll be brutally honest I don't think *Healthier Together* fits with anything. I think it's operating in a parallel universe … What we're lacking in GM is a Master Plan for the whole of GM which actually sets out the future shape of hospital provision. And what we're doing is … within each locality plan, carving it out across ten different places ten times, and I just think we need a really clear overview, where will the specialist hospitals be? You know, what would be the nature of them and we've just ducked it. (Local authority manager, ID62, February 2017; interview)

Bringing together new and existing elements was a non‐linear, conflict‐laden process. Given the relatively weak ties that held statutory organisations in the region together, tensions were continuously mitigated behind closed doors. Politically sensitive reconfigurations which risked rupturing consensus, not least the public scrutiny generated by major hospital reconfiguration could be strategically deferred. The assembling of the devolution project aligned different elements *as if* they were becoming a unified region moving forward together. To be seen to operate in unity was significant for solidifying the apparent togetherness of the region.

While the imagined unity of the assemblage was provisional, this had new material consequences. No single actor or organisation could determine relations in the region. The Partnership Team itself had a relatively limited capacity to force decisions across Greater Manchester:

Getting things done and holding people to account, [the Partnership Team] just don't have the lines of authority. You know, [the Chief Officer of the Partnership] has no direct authority over Foundation Trusts. The Health and Social Care Partnership has got no direct authority over councils. (NHS provider manager, ID74, July 2017; interview)

Despite all the individual NHS providers expressing initial support for devolution, given that they were not formal parties of the devolution agreement, they remained relatively autonomous with their own individual boards who agreed decisions on their individual finances. Work to ‘close the financial gap’ illustrates how remaking the regional whole was transforming relations between its constitutive parts. An animated debate surrounding moves to create a Greater Manchester‐wide financial control total, a mechanism to limit annual expenditure in the region, brought tensions to the fore given the impacts on the financial planning of seemingly discrete hospital organisations:

‘The spirit of togetherness on [a Greater Manchester wide] control total has some esoteric meaning but we all need to fight our own battles’ [ID54] … ‘it doesn't make sense to have a boundary around Greater Manchester’ [ID71] … ‘At the end of the day’, [ID66], insisted ‘the issue comes down to cash, that's what throws us into distress’. (NHS provider managers, February 2017; meeting observations)

While strengthening the boundary around Greater Manchester in an effort to limit and redirect healthcare expenditure towards primary care may have appealed to NHS commissioners, for NHS acute hospital providers, this was fundamentally transforming how they relate to one another. Their fears were that new elements such as a Greater Manchester‐wide financial control were being brought into and altering the initial aims for devolution that they initially, tentatively, agreed to.

Work by the Partnership Team and key political figures to persuade different local organisations to stay on‐board with the project *was* sufficient to sustain the project. This kept the region from falling apart despite its contradictions and conflicts. Reinforcing the coherence of the Greater Manchester project worked to discourage organisations ceasing involvement. Efforts were made to ensure that each locality received a ‘fair’ portion of the NHS transformation fund money. This held certain appeal for cash‐strapped local authorities. The potential for devolution to facilitate access to an increased proportion of national NHS capital for built and digital infrastructure remained an attractive prospect for NHS organisations. The combination of centrally driven austerity and frustrations with existing fragmented services helped create the conditions whereby there was seemingly ‘no alternative’:

I think if we're being very, very honest finance was probably at the heart of it. And I think there was recognition across the [local] economy that we couldn't carry on as we were … I think social care, to be fair, has been absolutely obliterated financially over the last few years and it was inevitable that health would pay for those social cuts because it just moves patients over. And I think we just reached the scenario where we had to do something. (CCG manager, March 2017, ID135; interview)

The above section incorporates just a few examples of work to reorganise health and care across the region and its localities and how this was successfully holding together as a meaningful, new territorial spatial project. However, as we now discuss, the apparent territorial coherence of Greater Manchester was distorted by seemingly distant actors and organisations becoming embedded within the regional assemblage attempting to shape and limit what was possible and connectedly, how local and regional actors sought to strategically rework these priorities to compete for new resources and powers.

## Renegotiating devolution and embedding regional agendas elsewhere

Devolution was often explained by managers in Greater Manchester as offering the possibility of *distancing* the region from the most overbearing centralising tendencies of national bodies. This would frequently be described in terms of getting national managers out of the region. For example:

… we could see what was coming and it [devolution] afforded an opportunity for us, if I put it quite bluntly … *to get NHS England off our pitch* … (Commissioning Manager, ID87, October 2016, interview; our emphasis)

Although a simplification, the imaginary of strengthening the boundaries of Greater Manchester to defend against national managers carried certain appeal. National managers suggested that they would indeed keep their distance, albeit on the condition that Greater Manchester proved it could work together as they required:

We will get off the pitch when results get better … [Greater Manchester] looks cohesive from afar … [but] there is one shot to make devolution work and you need to solve things altogether … can you prove that you're up for it? (National NHS manager, ID164, January 2016; meeting observations)

Resonating with Foucauldian‐inspired readings of power through notions of governmentality and governing‐at‐a‐distance, the implication was that managers in Greater Manchester would internalise national priorities and bring the region in line with the ‘centre’. In more dissenting moments, managers considered whether devolution was *distancing responsibility* from central government:

I think there's probably a growing inevitability, in part, I would say cynically it's about protecting Secretaries of State and ministers by insulating themselves by saying being able to point to when a crisis happens, to say actually that's yours, and what are you doing about it because you've now got the devolved authority and decision‐making and the money, so it's your problem. (NHS provider manager, ID71, August 2016; interview)

In this way, Greater Manchester was required to make politically contentious ‘tough decisions’ on behalf of central government at a time of austerity (Hammond *et al*. 2017).

At first sight, we might legitimately dismiss the devolution description as an exercise in political branding. It echoes previous reforms that adopted the language of the local to present the idea that governmental power has been devolved despite the continued influence of central government agendas (Moon and Brown 2000: 73). Hopes that Greater Manchester would be able to distance themselves through devolution, and the unwillingness to let this happen unless on the terms of national bodies is helpful for highlighting the limited extent of ‘devolution’ on offer. Whether governing‐at‐a‐distance or central government distancing responsibility, these spatial imaginaries would suggest national priorities were successfully embedded within the region without much resistance. While national politics of austerity were indeed embedded within the regional assemblage, as we shall illustrate in the rest of this section, relationships were more open‐ended than they might first appear.

As an evolving process, there was malleability to the interactions between Greater Manchester and the multitude of national intermediary arm's length bodies and agencies themselves grappling grappling with devolution and the formation of emerging local ‘accountable care organisations’ that were beginning to take shape:

I mean, [NHS England] have been brilliant in supporting this work, but all the regulators, NHSI, CQC, they need to come on the journey … That's not a criticism by the way. We need them to come on the journey, develop and understand some of these place‐based constructs, recognise that actually some of the models in which they operate are not fit for purpose. *They cannot inspect, all the time, discrete functions within discrete organisations because those things don't exist anymore*. (Local authority manager, ID52, May 2017; interview)

New integrated governance arrangements and alliances were increasingly at odds with existing national regulatory systems which still majored on individual organisational oversight. Undoubtedly, performance targets inherited from previous NHS reforms and new national ‘must dos’ were not displaced entirely once Greater Manchester became devolved. However, as assembled relationships evolved in the region, attempts by national health bodies to directly reach into the region to shape priorities were able to be strategically reworked to bolster their regional interests.

The ability of the region to be seen to work together as one remained critical for renegotiating national relationships. Prompt submission of annual National Planning documents for the entire region was one such case in point. Providing a Greater Manchester‐wide response, which included organisations not required to respond, was considered vital to the Partnership Team's attempts to secure new decision‐making capacity and resources. A senior manager relayed to the Partnership how their successful completion of planning documents, where others failed, was helping persuade national actors to further their support for the Greater Manchester project:

This is recognised nationally as an extraordinary achievement … we were, I think, maybe the only, or certainly one of the only areas in the country to do it, certainly at this scale, to achieve that position. And the great thing about it is the signal it sends … There had to be a few compromises, but we got over the message. The power, the totality of the message was really strong … a number of people picked up on it and fed back the impact it had. I know we're not here first and foremost to satisfy national bodies, but what it does is it unlocks goodwill about other things that we might need or want. (January 2017; meeting observation)

The Partnership Team produced its own Business Plan for the region in the months that followed. They even included elements of the Greater Manchester Mayor's manifesto commitments. Its purpose was to intensify the region's efforts to lobby nationally for greater devolution. Terms for brokering new arrangements were set out:

During this second year we will need even more devolved authority to make the changes required to achieve our objectives. For example, taking over further responsibilities from NHS England will support new commissioning arrangements, including for ambulance and NHS 111 services. We want to have more freedom in how we use money from the national *Better Care Fund* to join up health and social care services. And we aim to establish a single system control total for Greater Manchester finances. (Business Plan 2017/18: 8)

Previous work by the Partnership Team to move towards contentious Greater Manchester‐wide financial control totals were being enrolled into their current attempts to leverage new resources for the region. Their efforts were successful. Not only this, but the region managed to compete to gain access to limited national capital, despite NHS England rating overall progress in the region as ‘advanced’ rather than ‘outstanding’. This provided an additional funding source for the *Healthier Together* programme despite predating devolution.

Embedding regional priorities within central government went beyond the Department of Health. Making connections with the economic growth agenda of the wider devolution project, long‐standing efforts to bring together a renewed *Work and Health* programme sought to draw in a combination of new national and European Union funding:

We're negotiating hard with the Department for Work and Pensions (DWP) and the Treasury. However, DWP's national funding has reduced because of devolution and so we're in convocation with them, the European Social Fund can match DWP money invested. Anything that GM puts in above that makes DWP the junior partner and therefore devo becomes quite symbolic and changes the design, management and evaluation in GM. Removing of ring‐fences can greatly benefit the system with the individual at heart rather than being constrained by the national level. Therefore, we can start to develop a GM Work and Health Programme to fit the needs of GM rather than a national template and is an opportunity to test and learn in GM to help determine future decisions. (Combined Authority manager, ID14, May 2016; meeting observations)

New connections between health and work open up a whole set of political economic questions, not least the transformation of work and welfare under conditions of neoliberal hegemony in England. This is beyond the scope of the paper. Here, our concern is with the way in which regional actors were successful at forging new associations to mobilise and compete for resources independent of national NHS priorities.

The work of remaking health and care in Greater Manchester was not achieved by state and clinical actors alone. An extended circuit of think‐tanks and ‘global’ management consultants were drawn into Greater Manchester to legitimise and bolster, although not necessarily direct, regional priorities (Allen and Cochrane 2010). In fact, devolution brought about a whole new set of geographical connections. National and international delegates came to learn about health and care in Greater Manchester. The Partnership Team forged new ties with health managers in New York State, going on policy study tours to share ‘best practice’ and boost their profile internationally. Global pharmaceutical companies were courted to expand the life science industry in the region rather than the South East. Health think‐tanks held in high regard by central government were invited to observe meetings showcasing the region's developments. A positive assessment was circulated online and leaders were invited to provide keynote speeches at subsequent think‐tank events in London. These connections all helped bolster Greater Manchester's agenda on an increasingly international stage, presenting the region as a ‘pioneering’ integrated health and care system.

As our research drew to a close, the new Greater Manchester Mayor was collaborating with the Partnership Team to negotiate with central government for the region to pilot new social care reforms. Their attempt for new funds failed. Yet, Greater Manchester's lobbying was intensifying. As the Partnership would have it, this was all part of the ‘devolution journey’.

## Discussion and conclusion

In this paper, we have sought to illustrate how and why space is integral to the practices and politics of restructuring health and care and specifically how ideas of assemblage can help understand the remaking of a region. We described the work involved in remaking health and care through assembling different, often ill‐fitting elements that emerge, become ordered, mutate and fall apart, using the example of devolution in Greater Manchester, England. Our use of assemblage enabled us to highlight how health and care in Greater Manchester has been stabilised into a new devolved territorial formation *without becoming whole*, despite the search for, or claims to, its totality (Painter 2008). The ongoing political construction of the region is the outcome of the interplay of local, regional and national actors, organisations and agencies bringing together different policies and priorities, sometimes in conflict, at other times fitting together more easily. Our approach allowed us to demonstrate the multiple and diverse activities that contributed to bringing about relative coherence to the region despite the contradictory and conflicting dynamics involved in the restructuring of health and care systems and services.

The spatial reorganisation of health and care involves negotiation and contestation. An important contribution relates to the ways in which agency is conceived in assemblage thinking. While reference is often made to the significance of (system) leadership for major health and care reforms (Turner *et al*. 2016), our analysis demonstrates how agency is more usefully understood as embedded within the regional assemblage, rather than resulting from the actions of local, regional or national managers. Thinking about the governing of the regional assemblage moves us away from viewing national bodies as imposing top‐down control fixing the (national) ‘context’ for reform, by instead emphasising how different ‘national’ actors and agencies are embedded within the new territorial formation, more‐or‐less successfully shaping and limiting what is possible. Our data highlight how different national actors were embedded within the Greater Manchester devolution experiment, becoming part of the practices and politics of reassembling health and care. Central government‐led austerity measures were firmly embedded in the process. Yet, through drawing upon resources such as think‐tanks and management consultants independently, actors in Greater Manchester were mobilising to leverage their own agendas, to compete with other places in England for additional public resources during a period of austerity and to attract private pharmaceutical companies into the region.

Assemblage thinking helps us to pay attention to the evolving and sometimes unexpected dynamics of health and care reform. There is a tendency among evaluators of reform programmes to focus on comparing implementation in practice with the programme theory or logic model which underpins their design. This fails to address the more uncertain, emergent and political dimensions of the practices of working out and working through conflicts and crises. It also ignores the ways in which different groups conceive of changes from variegated positions which may or may not be mutually compatible. Rather than checking compliance against a shared mental model or explicit blueprint, researchers should investigate the conflict‐laden and political processes of assembling health and care systems comprised of multiple elements that hold together (or not) in ways that cut across different scales.

Where assemblage thinking has been used to understand practices and places of care, we view it as having significant application for examining the political reorganisation of health and care systems and services. This requires attention to how the socio‐spatial formation of health and social care is contested and shaped by the topological reach of different powers ‘beyond’ the boundaries of the region. It reminds us that reorganising health and care requires continuous political effort and that there is always the possibility for alternatives. Rather than approaching change in terms of before and after evaluations, we should examine the dynamic ways in which space is integral to reassembling and dismantling health and care as new policies and political actors are introduced and old ones fall away.

## Notes

1 For simplicity, we refer to Greater Manchester as a region. The full implications of restructuring health and care towards global competitive city‐regionalism are beyond the scope of this paper.

2 Since reduced through several mergers.
